# Quality and Readability of Four AI Chatbots Answering Search-Derived Patient Questions About Primary Aldosteronism: A Cross-Sectional Comparative Study

**DOI:** 10.3390/healthcare14183119

**Published:** 2026-09-21

**Authors:** Jianyu Chen, Zaihang Zhao, Shangyu Han, Jie Qin, Chenxia Wang, Yuxia Ma, Jianxun Cao

**Affiliations:** 1 School of Nursing, Lanzhou University, Lanzhou 730000, China; chjianyu2025@lzu.edu.cn (J.C.); 13255161008@163.com (Z.Z.); hanshy2025@lzu.edu.cn (S.H.); yuxiama@lzu.edu.cn (Y.M.); 2 Lanzhou University Hospital, Lanzhou University, Lanzhou 730000, China; qinjie@lzu.edu.cn; 3 Gansu Provincial Hospital, Lanzhou 730000, China; wang108abc@126.com

**Keywords:** artificial intelligence chatbot, primary aldosteronism, patient education, health information quality, readability, large language model

## Abstract

**Highlights:**

**What are the main findings?**

- Instrument-based information-quality scores differed among the tested chatbot configurations, but these differences do not establish greater factual accuracy, guideline concordance, or clinical superiority; the full-set DISCERN comparison was limited because most questions were not treatment-focused.
- Responses from all four chatbots exceeded the prespecified sixth-grade readability threshold.

**What are the implications of the main findings?**

- Chatbot responses should supplement, rather than replace, verified guidelines and individualized explanations from health professionals.
- Healthcare organizations should limit patient-facing chatbots to defined, low-risk uses. Organizations should also require source verification, plain-language output, human oversight, escalation advice, and monitoring after model updates.

**Abstract:**

**Background/Objectives:** Patients use artificial intelligence (AI) chatbots for medical information, but response quality, source transparency, and readability for primary aldosteronism (PA) remain uncertain. This study compared four chatbot configurations and assessed whether their responses met patient-education reading levels. **Methods:** During a single-time-point snapshot on 27 July 2026, 17 questions derived from Medical Subject Headings and five-year worldwide Google Trends data were submitted once to GPT-5.5 through ChatGPT Plus, Copilot in Smart mode, Gemini 3.5 Flash, and the default Perplexity model, yielding 68 responses. DISCERN, the Ensuring Quality Information for Patients (EQIP) instrument, the Journal of the American Medical Association (JAMA) benchmarks, and the Global Quality Score (GQS) assessed structural information quality; six formulas assessed readability. These instruments did not assess statement-level factual accuracy. Differences were examined using Friedman tests and paired Wilcoxon signed-rank tests with Holm adjustment. **Results:** Scores differed for DISCERN (χ^2^ = 16.717, *p* < 0.001), EQIP (χ^2^ = 31.125, *p* < 0.001), JAMA (χ^2^ = 48.851, *p* < 0.001), and GQS (χ^2^ = 9.874, *p* = 0.020). Copilot had the highest median DISCERN and EQIP scores (43.00 and 75.00), followed by Perplexity (42.00 and 70.00). Median JAMA scores were 0 for ChatGPT and Gemini and 1 for Copilot and Perplexity. These instrument-based differences do not establish greater factual accuracy, guideline concordance, or clinical superiority; moreover, the full-set DISCERN comparison has limited interpretability because only two questions were explicitly treatment-related. Item-level kappa values ranged from 0.844 to 0.924, and total-score ICC(2,1) values ranged from 0.846 to 0.883. No readability measure met the sixth-grade benchmark. **Conclusions:** In these configurations, chatbots produced PA information. However, source transparency remained low, and language was overly complex. Relative score differences should not be interpreted as factual superiority. Patient-facing use requires verified sources, scope limits, human oversight, and routes to care.

## 1. Introduction

Primary aldosteronism (PA) is endocrine hypertension caused by autonomous or relatively autonomous aldosterone secretion from one or both adrenal glands. Excess aldosterone causes renal sodium retention, volume expansion, and high blood pressure; severe cases may also cause hypokalemia. At similar blood pressure levels, patients with PA have higher risks of stroke, coronary heart disease, atrial fibrillation, heart failure, and kidney damage than those with essential hypertension. Timely recognition and aldosterone-targeted medical or surgical treatment can improve blood pressure control and reduce these risks [1,2].

Patient education about PA must explain more than high blood pressure. Patients may need to understand how aldosterone relates to renin, why hypokalemia and metabolic alkalosis occur, how primary and secondary hyperaldosteronism differ, and how screening leads to confirmation, subtype classification, and treatment. Adrenal venous sampling, bilateral adrenal hyperplasia, glucocorticoid-remediable aldosteronism, and spironolactone adverse effects are technically demanding concepts. Incomplete information, excessive certainty, or unclear treatment eligibility may impair patients’ understanding of further testing, medication monitoring, and surgery.

ChatGPT, Gemini, Perplexity, and Copilot can give immediate conversational disease explanations and reduce the effort of conventional web searches. Evaluations of chatbot responses about sexually transmitted diseases, maintenance hemodialysis, palliative care, and public health questions have found variation in information quality, source transparency, and readability. Most generated texts also exceeded the recommended reading level for patient education materials [3,4,5,6].

Standardized multi-model comparisons of responses to search-derived patient questions about PA remain scarce. PA is often missed, and its diagnostic and treatment pathways require context [7]. These characteristics make PA a useful test of AI-mediated health communication. Following Chatbot Assessment Reporting Tool (CHART) principles, we used real-world search interest to compare the quality, transparency, educational value, and readability of PA information from four AI chatbots. We asked whether these dimensions differed among models and whether the language met recommended reading standards for public-facing patient education [8].

## 2. Materials and Methods

### 2.1. Study Design

This web-based cross-sectional comparative study was conducted online on 27 July 2026 in Lanzhou, Gansu Province, China. English was used, and the geographic scope of Google Trends was set to worldwide. Only publicly available search trends and AI-generated text were analyzed; no patient records or personally identifiable information were used. The protocol was designed to capture the first answer that a patient might receive at one specified time, rather than to estimate within-model repeatability. Study design and reporting followed CHART recommendations for model identification, prompt derivation, response collection, repeatability control, evaluation measures, and statistical analysis [8].

### 2.2. Development of Search-Derived Patient Questions

PA terminology was first verified in the Medical Subject Headings database. Worldwide related queries and search terms from the previous 5 years (2021–2026) were then retrieved from Google Trends using “hyperaldosteronism” as the base search term. Google Trends aggregates different languages and expressions under one concept, which helps identify sustained public interest in health topics. It does not provide exact search counts or user demographic characteristics [9]. The term ‘hyperaldosteronism’ was retained as the prespecified Google Trends entry term used to generate the related-query set. Clinically, PA refers to autonomous aldosterone excess, whereas hyperaldosteronism is a broader term that can include secondary forms. The questions retained public search wording, while PA was used for the target disorder in the manuscript. Google Trends does not establish actual patient question frequencies or individual information needs.

The initial search produced 34 candidate items. Two raters independently reviewed them and excluded 3 unrelated items and 14 duplicates or highly similar items. Seventeen core questions remained. The raters converted the search expressions into complete, clear English questions from a patient perspective for direct chatbot entry. They improved grammar and usability without changing search intent and preserved the link between each original term and standardized question.

### 2.3. Selection of AI Chatbots and Response Collection

The four platforms were selected a priori because they were prominent consumer-facing chatbot services accessible through standard web interfaces, used different technical approaches, and could answer patient-style health questions without API-level customization. The tested configurations were ChatGPT using GPT-5.5 through a ChatGPT Plus account (OpenAI, San Francisco, CA, USA; Memory and Custom Instructions disabled), Gemini 3.5 Flash through a free account (Google LLC, Mountain View, CA, USA), Perplexity using the default model through a signed-in free account (Perplexity AI, San Francisco, CA, USA), and Copilot using Smart mode through a signed-in free account (Microsoft Corporation, Redmond, WA, USA). Within each platform, the specified configuration was chosen pragmatically because it was the standard or directly accessible consumer option available to the study account on the collection date. The selection was not based on evidence that these were the most frequently used patient-facing versions, and the configurations were not intended to represent the highest-capability or all available versions of each product. Neither Copilot nor Perplexity displayed a more specific underlying model identifier in the interfaces used on the data-collection date. All four received identical English wording for each question. Each question was submitted once in a new conversation on 27 July 2026, and the first response was retained to represent an unrefined, one-time user interaction. No follow-up prompts, rewriting requests, or manual edits were used, and all other platform settings remained at their defaults. The final dataset comprised 17 × 4 = 68 original responses.

The complete original responses, including titles, paragraph structure, in-text citations, links, and source lists, were used for information-quality assessment so that transparency features remained available. Readability was assessed in a separate preprocessed version. Text was converted to plain form; bullets, numbering, URLs, in-text citation markers, and standalone reference lists were removed. Content about disease mechanisms, risks, testing, and treatment was retained.

### 2.4. Information-Quality Assessment Tools

DISCERN was used to evaluate the reliability of written consumer health information and information about treatment choices. It assesses whether aims are clear, sources are explicit, information is balanced, and the benefits and risks of treatment options are presented. Under the prespecified categories used in this study, scores of 63–75 were considered excellent, 51–62 good, 39–50 fair, 27–38 poor, and 16–26 very poor [10].

DISCERN does not provide a separate ‘not applicable’ response option. When a response did not address a treatment-related criterion, the item was scored as 1, indicating that the criterion was not fulfilled.

EQIP assesses patient education materials for completeness, presentation, and usability. The 20-item instrument assigns 1 point for “yes” and 0 points for “no”; the final score was calculated as (total score/20) × 100. Scores of 76–100 were considered excellent, 51–75 good, 26–50 poor, and 0–25 indicative of serious quality problems [11].

The Global Quality Score (GQS) rates overall information quality, organization, flow, and practical value to patients on a 5-point scale, where 1 indicates very poor quality, and 5 indicates excellent quality [12].

The JAMA benchmarks assess authorship, attribution, disclosure, and currency. Each domain is scored 0 or 1 for a total score of 0–4. In this study, JAMA was used only as a structural indicator of traceability and source transparency. A high JAMA score does not establish that a medical claim is correct, and a low score does not establish that it is incorrect [13].

These instruments cover treatment-information quality, the content and presentation of educational materials, overall usefulness, and source transparency. They were developed mainly for static written materials and do not fully capture conversational follow-up or adaptation after user clarification. They are collectively described as information-quality measures and do not replace statement-level fact-checking, guideline-concordance assessment, or evaluation of potential clinical harm.

### 2.5. Rating Procedure

Two raters with medical backgrounds independently scored all responses. Before formal assessment, a random sample of approximately 10% of responses was used to align item interpretation and scoring boundaries. Materials were randomly coded, and raters were blinded to the source chatbot. All scoring discrepancies were reviewed and adjudicated by a third senior evaluator before the consensus scores were finalized. Inter-rater agreement before adjudication was assessed using unweighted Cohen’s kappa for binary EQIP and JAMA item ratings, quadratic-weighted Cohen’s kappa for ordinal DISCERN item ratings and GQS, and a two-way random-effects, absolute-agreement, single-measure intraclass correlation coefficient [ICC(2,1)] for each total score. Because citation links and source-list formats were retained for transparency scoring, distinctive presentation features could still have suggested the generating platform; successful blinding was not formally tested.

### 2.6. Readability Assessment

English-language responses were assessed with the Automated Readability Index (ARI), Flesch Reading Ease Score (FRES), Gunning Fog Index (GFI), Flesch-Kincaid Grade Level (FKGL), Coleman-Liau Index (CL), and Simple Measure of Gobbledygook (SMOG) Index. Except for FRES, each score approximates the educational grade required to understand a text; higher FRES values indicate easier reading [14,15].

A sixth-grade level was retained as the prespecified benchmark because public-facing health information should be accessible to readers with limited health literacy, consistent with the audience-centered approach described in the National Institutes of Health (NIH) Clear and Simple guidance [16]. It was treated as a demanding communication target rather than a disease-specific pass/fail standard. For interpretation, an eighth-grade level was also considered as a more permissive contextual benchmark. FRES ≥ 80 and ARI, GFI, FKGL, CL, and SMOG < 6 were considered to meet the prespecified benchmark. Unavoidable PA terminology can raise formula-based estimates even when a term is explained in plain language, and SMOG is particularly sensitive to polysyllabic words. Readability was calculated with Python 3.10.4 and textstat 0.7.13.

### 2.7. Statistical Analysis

The unit of analysis was one chatbot response to one question. Continuous variables were assessed for distribution and summarized as median and interquartile range [median (Q1, Q3)]. Because the same 17 questions were submitted to all four chatbots, each question was treated as the matched block. DISCERN, EQIP, JAMA, and GQS values were compared using the Friedman test, with Kendall’s W reported as the overall effect size. When an overall test was significant, paired Wilcoxon signed-rank tests were performed and adjusted for six pairwise comparisons using the Holm method. A descriptive sensitivity analysis restricted DISCERN to the two explicitly treatment-related questions (Questions 13 and 14); because only two observations were available per chatbot, no inferential test was performed for this analysis. Readability scores were summarized as medians and interquartile ranges and interpreted descriptively against the prespecified thresholds. Two-sided *p* < 0.05 was considered statistically significant.

## 3. Results

### 3.1. Core Questions and Dataset

In total, 17 search-derived patient questions about PA remained after unrelated and duplicate items were removed from the 34 candidates. The questions covered definitions and subtypes, causes and symptoms, effects on blood pressure and potassium, aldosterone and renin, diagnosis, treatment, spironolactone, bilateral adrenal hyperplasia, a hereditary subtype, and metabolic alkalosis (Table 1). The four chatbots generated 68 responses.

### 3.2. Overall Comparison of Information Quality

DISCERN, EQIP, JAMA, and GQS values differed significantly among the four chatbots (Table 2). Copilot had the highest median DISCERN score at 43.00 (39.00, 46.00), which corresponds to fair quality. Perplexity scored 42.00 (37.00, 44.00), also in the fair range. ChatGPT and Gemini scored 33.00 (33.00, 40.00) and 36.00 (32.00, 39.00), respectively, and were classified as poor overall.

All four median EQIP scores fell in the good category. Copilot had the highest median [75.00 (70.00, 75.00)], followed by Perplexity [70.00 (60.00, 80.00)]. Median GQS values were 4 for ChatGPT, Copilot, and Perplexity and 3 for Gemini. Median JAMA scores were 1 for Copilot and Perplexity and 0 for ChatGPT and Gemini. Even the better-performing chatbots rarely reported authorship, sources, disclosures, or currency.

Before adjudication, item-level inter-rater agreement was strong: quadratic-weighted kappa was 0.924 for DISCERN and 0.844 for GQS, while unweighted kappa was 0.879 for EQIP and 0.917 for JAMA. Total-score ICC(2,1) values were 0.883 (95% CI, 0.817–0.926) for DISCERN, 0.871 (0.799–0.919) for EQIP, 0.856 (0.771–0.910) for JAMA, and 0.846 (0.762–0.902) for GQS.

**Figure 1:**
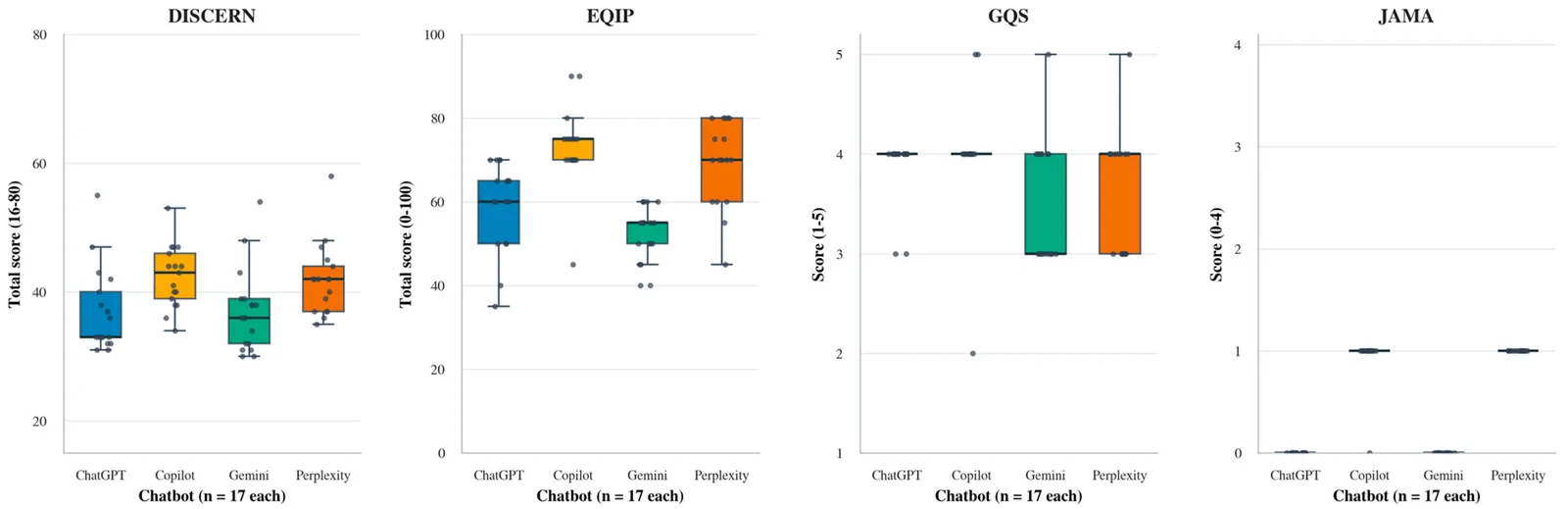
Distributions of DISCERN, EQIP, GQS, and JAMA scores across the four AI chatbots. Points are question-level responses (n = 17 per chatbot). Boxes show medians and interquartile ranges, and whiskers extend to 1.5 times the interquartile range.

JAMA had the largest matched-model effect (Kendall’s W = 0.958), followed by EQIP (W = 0.610), DISCERN (W = 0.328), and GQS (W = 0.194). The overall Friedman tests were significant for DISCERN (chi-square = 16.717, *p* < 0.001), EQIP (chi-square = 31.125, *p* < 0.001), JAMA (chi-square = 48.851, *p* < 0.001), and GQS (chi-square = 9.874, *p* = 0.020). Figure 1 shows the question-level score distributions.

### 3.3. Post Hoc Pairwise Comparisons

In paired Wilcoxon signed-rank comparisons with Holm adjustment (Table 3), Copilot and Perplexity scored higher than Gemini for DISCERN (*p* = 0.015 and *p* = 0.008, respectively); no other DISCERN comparison remained significant. For EQIP, Copilot scored higher than ChatGPT (*p* = 0.005) and Gemini (*p* = 0.002), while Perplexity scored higher than ChatGPT (*p* = 0.027) and Gemini (*p* = 0.003). Copilot and Perplexity both scored higher than ChatGPT and Gemini for JAMA (all *p* < 0.001); Copilot did not differ from Perplexity, and ChatGPT did not differ from Gemini. Although the overall GQS test was significant, none of the six Holm-adjusted pairwise comparisons was significant.

In the descriptive sensitivity analysis restricted to the two explicitly treatment-related questions, the median DISCERN scores were 49.0 (range, 43–55) for ChatGPT, 48.5 (44–53) for Copilot, 51.0 (48–54) for Gemini, and 53.0 (48–58) for Perplexity. Given the small number of questions, these findings were interpreted descriptively and were not subjected to inferential testing. Because 15 of the 17 questions were not explicitly treatment-related, the full-set DISCERN comparison was numerically dominated by prompts for which some treatment-specific criteria were not relevant; it should therefore be interpreted cautiously.

### 3.4. Readability

**Figure 2:**
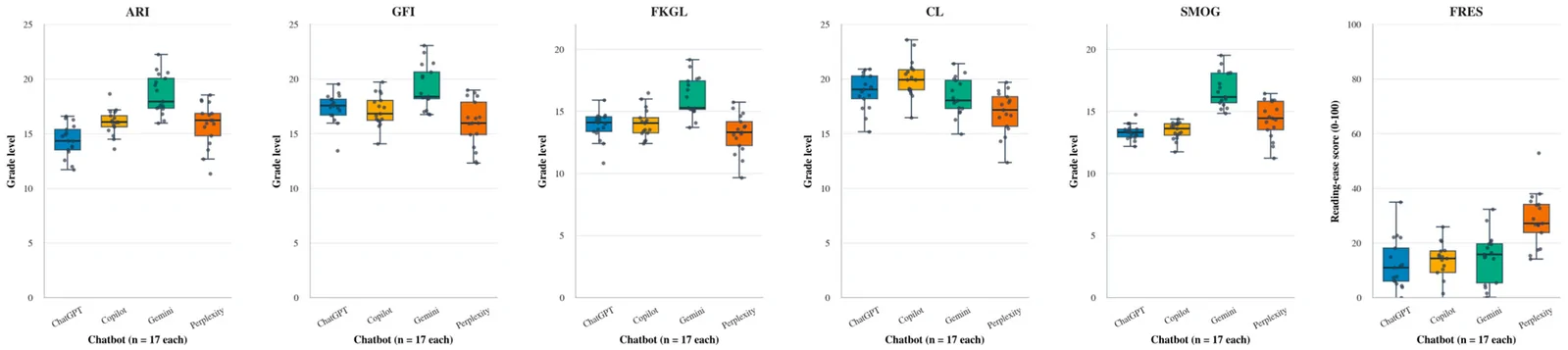
Distributions of six readability measures across the four AI chatbots. Points are question-level responses (n = 17 per chatbot). Boxes show medians and interquartile ranges, and whiskers extend to 1.5 times the interquartile range. ARI, GFI, FKGL, CL, and SMOG are grade-level estimates; FRES is a reading-ease score.

All four chatbots produced text above the sixth-grade level (Table 4 and Figure 2). Across models, median ARI values ranged from 14.32 to 17.93, GFI from 15.97 to 18.39, FKGL from 13.31 to 15.28, CL from 17.18 to 19.94, and SMOG from 13.30 to 16.16; all exceeded the acceptable threshold of 6. Median FRES values ranged from 10.88 to 27.08, below the recommended threshold of 80. The practical conclusion was evident from the descriptive statistics alone, as all median grade estimates were far above 6 and all FRES medians were well below 80. Even under the more permissive eighth-grade contextual benchmark, all median grade-level estimates remained above the target range.

Model rankings differed among formulas. Gemini had the highest median ARI, GFI, FKGL, and SMOG values, while Copilot had the highest CL value. Perplexity had the highest FRES and the lowest GFI, FKGL, and CL values; these scores corresponded to easier reading on several dimensions. ChatGPT had the lowest ARI and SMOG values. Yet none of the chatbots approached the target level for patient education, so relative advantages did not indicate adequate readability.

### 3.5. Question-Level Heterogeneity

**Figure 3:**
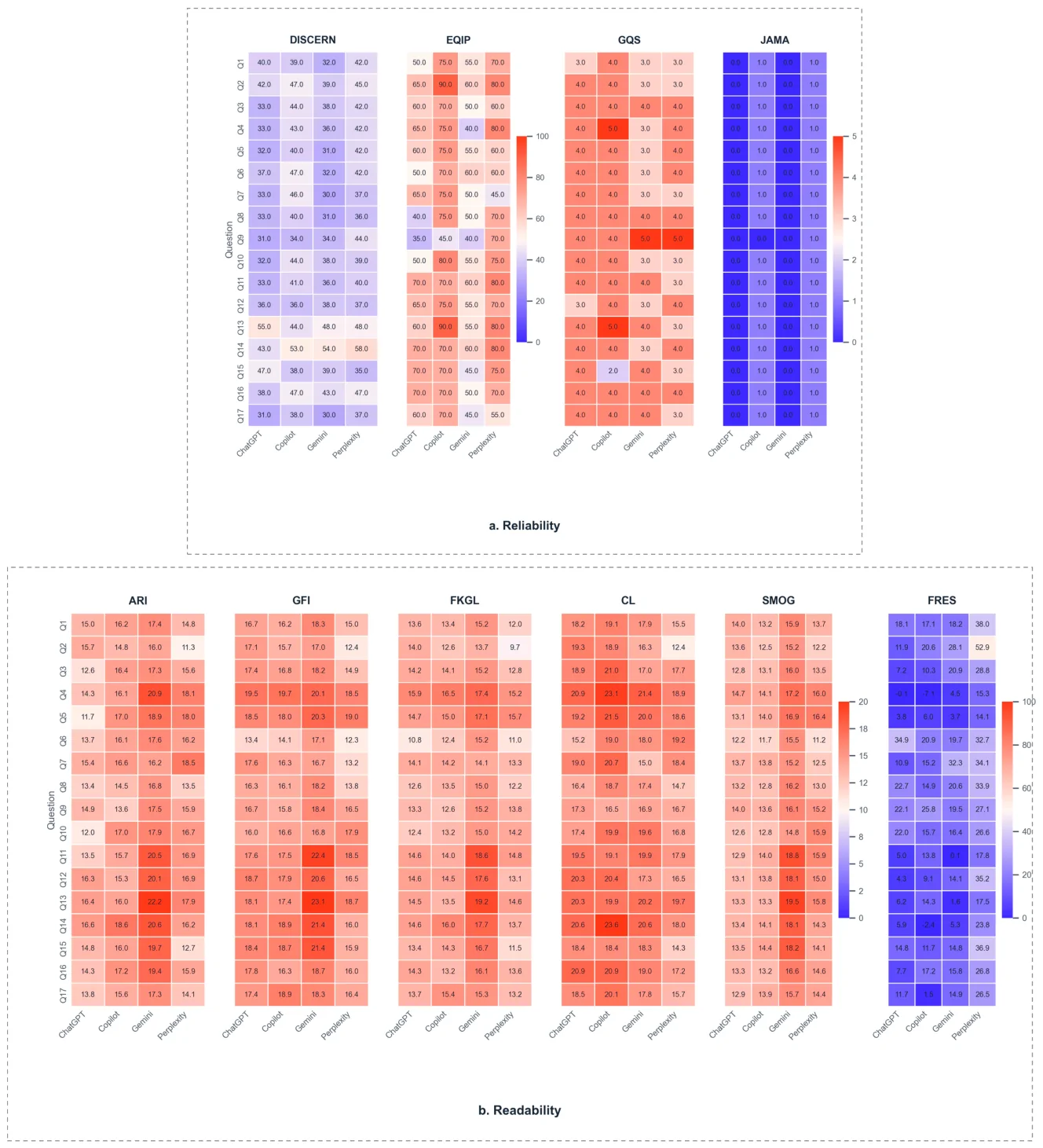
Enlarged question-level heat maps of information quality and readability across the four AI chatbots. (**a**) DISCERN, EQIP, GQS, and JAMA. (**b**) ARI, GFI, FKGL, CL, SMOG, and FRES. Rows represent the 17 questions, and columns represent the chatbots.

Quality and readability varied across questions within each chatbot (Figure 3). Model rankings also changed by question. Copilot and Perplexity performed relatively well for DISCERN, EQIP, and JAMA on most questions, although other chatbots achieved similar or higher overall quality scores for individual questions. Responses about diagnostic mechanisms and hereditary subtypes generally used more technical vocabulary; the same was true for metabolic abnormalities. Readability varied accordingly. A single overall mean or median therefore cannot represent performance for a specific patient question.

## 4. Discussion

### 4.1. Principal Findings

Across the 17 search-derived patient questions about PA, the four chatbots differed in DISCERN, EQIP, JAMA, and GQS values. Copilot and Perplexity scored relatively well on several measures, but the highest median DISCERN score was only fair, and the highest median JAMA score was 1 of 4. All responses exceeded the sixth-grade reading level, and each model’s performance varied by question. Complete, well-organized responses may still lack sources, contain inaccuracies, or be too difficult for patients.

### 4.2. Between-Model Differences and Absolute Limitations

Copilot and Perplexity had their clearest relative advantages in DISCERN, EQIP, and JAMA. Platforms with integrated web retrieval or visible citations may include more source links and structured sections. These features can raise traceability and organization scores, including those for treatment-option presentation. Studies of sexually transmitted diseases and maintenance hemodialysis similarly found that Perplexity or Copilot scored relatively well on these measures and linked this pattern to citation mechanisms and structured presentation [3,4]. This apparent advantage should be interpreted as an architecture- and interface-dependent advantage in traceability, not as evidence of superior factual accuracy. Procurement decisions should therefore assess retrieval capability together with link validity, source authority, recency, and consistency between each claim and its cited source.

No chatbot’s absolute scores support its use as a reliable substitute for professional information. Copilot and Perplexity had median JAMA scores of only 1; authorship, disclosure, and currency were usually absent even when a source appeared. JAMA alone cannot establish link validity or source authority. Nor can it show whether a citation supports a specific claim. ChatGPT and Gemini had a median JAMA score of 0, so users may be unable to judge provenance or recency from the response.

PA information requires balanced communication. The 2025 Endocrine Society guideline states that screening, suppression testing, subtype evaluation, medical therapy, and surgery depend on patient preferences and available resources [2]. Unilateral and bilateral disease follow different treatment paths; spironolactone requires blood pressure and laboratory monitoring, including serum potassium and kidney function. A fluent chatbot can still score poorly on DISCERN if it gives one simplified conclusion without explaining prerequisites, alternatives, and uncertainty. DISCERN also requires caution when it is applied to questions about definitions or mechanisms. Such responses may receive low treatment-item scores because treatment content is not relevant to the prompt. The analysis restricted to Questions 13 and 14 was therefore used as a descriptive sensitivity check; with only two questions, it could not support inferential comparisons between models.

Because 15 of the 17 questions did not explicitly concern treatment, the full-set DISCERN findings were largely determined by non-treatment prompts and should not be interpreted as a definitive comparison of overall clinical information quality.

### 4.3. Readability and Implications for Patient Education

Readability was inadequate across all models. Median grade-based scores generally corresponded to late secondary school or university education, while FRES values were in the very difficult range. PA responses necessarily include multisyllabic terms such as hyperaldosteronism, hypokalemia, renin, adrenal hyperplasia, and glucocorticoid-remediable aldosteronism. Long sentences, subordinate clauses, and dense mechanistic explanations increase the estimated difficulty. Gemini was the most difficult on several metrics, which suggests a more technical or academic style. Although Perplexity was easier to read, it remained far from the sixth-grade standard. The sixth-grade threshold should therefore be interpreted as a demanding plain-language target rather than proof that every PA term can be reduced to sixth-grade vocabulary.

Previous studies report the same pattern. Yıldız and Söğütdelen found that none of four chatbots providing information about sexually transmitted diseases met a sixth-grade reading level. Cao et al. compared five models on maintenance hemodialysis and found high ARI, GFI, FKGL, CL, and SMOG scores and low FRES values across all models [3,4].

Readability formulas measure word length, sentence length, and the proportion of complex words; they do not measure patient comprehension or content accuracy [14,15]. Agreement across formulas still points to a communication barrier. Low health literacy is associated with poorer health outcomes. Complex materials may cause patients to misunderstand screening and medication monitoring. They may also treat general information as individualized medical advice [17]. SMOG may yield comparatively high grade estimates in this setting because it gives substantial weight to polysyllabic medical terms. The study did not compare the chatbot responses with matched patient materials from NIH, MedlinePlus, or professional societies; it therefore cannot determine whether the chatbots were more complex than the topic itself requires.

Prompts and system instructions could require sixth-grade vocabulary and one idea per sentence. They could also request plain-language definitions at first use and a list of situations requiring medical care. Studies suggest that large language models can reduce the linguistic complexity of patient materials when given specific simplification instructions. Clinical review remains necessary because simplification may remove important risk information [18,19].

### 4.4. Potential Leadership and Governance Considerations

The following considerations are interpretive, practice-oriented recommendations informed by the observed readability burden and source-transparency limitations; they were not directly evaluated as outcomes in this study.

Healthcare organizations should define permitted and prohibited uses before embedding a general-purpose chatbot in a patient-facing pathway. A multidisciplinary oversight group should approve use cases, specify when human review is required, assign responsibility for content and incident management, and suspend unsafe functions. Low-risk uses may include drafting questions or reinforcing clinician-reviewed education, whereas diagnosis, triage, medication changes, and treatment selection should not be delegated to an unsupervised chatbot [20].

Deployment decisions can be guided by four questions: What is the consequence of an incorrect answer? Is the query time-sensitive? Does it require patient-specific data? Can important claims be traced to current authoritative sources? Low-consequence, non-personalized tasks may be considered with source and scope disclosures; moderate-risk tasks require professional review; and high-consequence or time-sensitive queries should be routed to clinical contact. In patient portals, queries involving severe symptoms, medication changes, or surgery should trigger an escalation pathway rather than an autonomous recommendation.

Procurement should assess source provenance, update control, claim-source consistency, plain-language options, accessibility, privacy, audit logs, incident reporting, and the ability to disable high-risk functions, rather than cost or aggregate scores alone. The relative traceability advantage of Copilot and Perplexity does not ensure that cited sources are authoritative, current, or correctly linked to claims.

After deployment, organizations should repeat a small sentinel set of representative and high-risk questions after model updates and review complaints, near misses, and subgroup performance. The most important action for leaders in the next 12 months is an institution-wide policy that assigns accountability and requires risk classification, human review for consequential content, escalation pathways, and post-deployment monitoring [20].

### 4.5. Ethical, Legal, and Social Implications

Embedding a chatbot in an institutional website or portal may reasonably be interpreted by patients as organizational endorsement. Institutions should therefore state the tool’s scope, limitations, source-update date, and route for reporting harmful content, and should identify who is responsible for approval, review, and incident response. A disclaimer alone does not define professional or institutional responsibility; accountability should be assigned according to the use context and applicable law. AI-generated information should support, not replace, informed consent and shared decision-making. Clinicians also need time and communication training to correct misinformation without dismissing the patient’s concerns or damaging trust [21].

Bias and unequal access remain relevant even when a response is fluent. Performance may differ by language, health literacy, disability, age, or the way a question is phrased. Public chatbots may also invite patients to enter sensitive information. Governance policies should prohibit unnecessary disclosure of identifiable data, assess subgroup performance, provide accessible alternatives, and avoid making a chatbot the only route to information or care.

### 4.6. Implications for Clinical Communication and Platform Design

Patients may use chatbots to draft questions or review concepts already explained by a health professional. They should not use them to stop medication, change a spironolactone dose, decline suppression testing, or select surgery without professional advice. Clinicians should ask about AI use when relevant, correct misconceptions, provide reviewed plain-language materials, and use teach-back for difficult concepts such as the aldosterone-to-renin ratio and adrenal venous sampling.

Platforms should offer tiered reading options, define technical terms at first use, present one actionable idea at a time, display dated sources, and distinguish general education from individualized advice. High-risk responses should state uncertainty and include a clear recommendation to seek professional care. Simplification still requires clinical review because removing technical details can also remove safety information.

Model developers and healthcare deployers should use dynamic verification rather than a one-time approval. Testing should be repeated after changes to the model, retrieval system, or interface, and failures should be analyzed at the question and claim level. These controls require designated staff, an audit schedule, and a documented route from detected failure to correction.

### 4.7. Strengths and Limitations

Question development combined standardized terminology with real-world search trends. All four chatbots received identical questions under a standardized collection process. DISCERN, EQIP, GQS, and JAMA measured complementary aspects of information quality; six formulas assessed readability. The analyses corrected for multiple comparisons, reported effect sizes, and displayed question-level heat maps.

This study was a single-time-point snapshot of four configurations on 27 July 2026. Each question was submitted once, so within-model variability across repeated prompts was not estimated. Some pairwise differences may therefore reflect stochastic response variation as well as stable differences between systems. The findings should not be generalized to later model updates, other account types, or other configurations within the same product family; in particular, results for Gemini 3.5 Flash do not represent all Gemini models.

Question development used one Google Trends entry term, ‘hyperaldosteronism.’ Google Trends provides aggregated related queries rather than actual patient-level needs or reliable question frequencies, and alternative terms, such as ‘primary aldosteronism’, ‘Conn syndrome’, and ‘adrenal adenoma’, were not evaluated as separate entry terms. The question set may therefore omit other patient concerns. Only English prompts and default or specified platform modes were tested; multilingual responses, prompt engineering, simplified-language instructions, and comparison with matched patient materials from authoritative organizations were outside the study protocol.

DISCERN, EQIP, GQS, and JAMA were developed mainly for static written materials. They permit structured comparison but do not fully assess interactive dialogue, statement-level factual accuracy, guideline concordance, or potential clinical harm. The raters had medical backgrounds but were not recruited as PA subspecialists, and no separate blinded specialist accuracy review was performed. Citation style may also have partly revealed chatbot identity despite random coding. Because DISCERN is primarily treatment-focused, applying the full instrument to non-treatment questions may underestimate their information quality when treatment-specific criteria are not relevant; moreover, the treatment-only sensitivity analysis included only two questions. Readability formulas were not validated against comprehension or behavioral outcomes in patients with PA. Good-to-strong agreement between raters reduces, but does not remove, judgment inherent in these instruments.

Taken together, the single-response design, configuration-specific snapshot, treatment-focused DISCERN instrument, and single-term search strategy limit the generalizability and interpretability of the findings, especially the pairwise model comparisons.

To evaluate clinical reliability beyond structural quality, future studies should create a prespecified reference standard based on current PA guidance; have at least two PA specialists, blinded to chatbot identity, independently rate claim-level accuracy, completeness, guideline concordance, potentially harmful omissions or advice, and citation support; adjudicate disagreements; and report inter-rater agreement. Repeated prompts across independent sessions and comparison with vetted patient materials are also needed. Patient comprehension, subsequent health behavior, effects on clinician workload, and performance across languages and health-literacy groups are also needed before a leadership mandate for routine deployment can be supported.

## 5. Conclusions

In this single-time-point comparison of the tested configurations, information-quality scores differed among chatbots, but relative advantages in structure or source visibility should not be interpreted as superior factual accuracy. Source transparency remained limited, and all responses exceeded the prespecified plain-language benchmark. General-purpose chatbots may support low-risk patient education under defined conditions, but they cannot replace clinical guidelines, claim-level verification, or individualized communication with health professionals. Healthcare organizations should define permitted uses, require human review for clinically consequential content, provide escalation routes, and monitor performance after model updates.

## Figures and Tables

**Table 1:** Worldwide Search-Derived Patient Questions About Primary Aldosteronism, 2021–2026.

No.	Question
1	What is hyperaldosteronism?
2	What is primary hyperaldosteronism?
3	What is secondary hyperaldosteronism?
4	What is the difference between primary and secondary hyperaldosteronism?
5	What causes hyperaldosteronism?
6	What are the symptoms of hyperaldosteronism?
7	Can hyperaldosteronism cause high blood pressure?
8	How does hyperaldosteronism affect potassium levels?
9	Why can hyperaldosteronism cause hypokalemia?
10	What is aldosterone and how is it related to hyperaldosteronism?
11	What is the role of renin in diagnosing hyperaldosteronism?
12	How is hyperaldosteronism diagnosed?
13	How is hyperaldosteronism treated?
14	How is spironolactone used to treat hyperaldosteronism?
15	What is bilateral adrenal hyperplasia?
16	What is glucocorticoid-remediable aldosteronism?
17	What is metabolic alkalosis and how is it related to hyperaldosteronism?

**Table 2:** Information-Quality Scores of the Four AI Chatbots.

Measure	ChatGPT	Copilot	Gemini	Perplexity	W	*p*
DISCERN	33.00 (33.00, 40.00)	43.00 (39.00, 46.00)	36.00 (32.00, 39.00)	42.00 (37.00, 44.00)	0.328	<0.001
EQIP	60.00 (50.00, 65.00)	75.00 (70.00, 75.00)	55.00 (50.00, 55.00)	70.00 (60.00, 80.00)	0.610	<0.001
JAMA	0.00 (0.00, 0.00)	1.00 (1.00, 1.00)	0.00 (0.00, 0.00)	1.00 (1.00, 1.00)	0.958	<0.001
GQS	4.00 (4.00, 4.00)	4.00 (4.00, 4.00)	3.00 (3.00, 4.00)	4.00 (3.00, 4.00)	0.194	0.020

Data are presented as median (Q1, Q3). *p* values were obtained using the Friedman test; Kendall’s W is the Friedman effect size. GQS, Global Quality Score.

**Table 3:** Pairwise Comparisons of Quality Scores Based on Paired Wilcoxon Signed-Rank Tests (Holm-Adjusted *p* Values for six comparisons).

Model Comparison	DISCERN	EQIP	JAMA	GQS
ChatGPT-Copilot	0.059	0.005	<0.001	0.959
ChatGPT-Gemini	1.000	0.092	1.000	0.203
ChatGPT-Perplexity	0.051	0.027	<0.001	0.287
Copilot-Gemini	0.015	0.002	<0.001	0.261
Copilot-Perplexity	1.000	0.158	0.635	0.261
Gemini-Perplexity	0.008	0.003	<0.001	0.959

**Table 4:** Readability Scores of the Four AI Chatbots.

Measure	ChatGPT	Copilot	Gemini	Perplexity	Sixth-Grade Threshold
ARI	14.32 (13.52, 15.39)	16.06 (15.62, 16.64)	17.93 (17.34, 20.05)	16.21 (14.81, 16.85)	<6
GFI	17.57 (16.70, 18.14)	16.83 (16.21, 18.04)	18.39 (18.19, 20.63)	15.97 (14.90, 17.89)	<6
FKGL	14.10 (13.37, 14.55)	14.04 (13.25, 14.48)	15.28 (15.15, 17.44)	13.31 (12.24, 14.16)	<6
CL	19.03 (18.19, 20.27)	19.94 (19.01, 20.86)	18.03 (17.30, 19.88)	17.18 (15.68, 18.37)	<6
SMOG	13.30 (12.93, 13.56)	13.61 (13.08, 13.99)	16.16 (15.68, 18.08)	14.44 (13.51, 15.80)	<6
FRES	10.88 (5.92, 18.05)	14.28 (9.09, 17.05)	15.77 (5.29, 19.68)	27.08 (23.80, 34.06)	≥80

Data are presented as median (Q1, Q3). ARI, Automated Readability Index; GFI, Gunning Fog Index; FKGL, Flesch-Kincaid Grade Level; CL, Coleman-Liau Index; SMOG, Simple Measure of Gobbledygook; FRES, Flesch Reading Ease Score.

## Data Availability

The 17 questions and summary results are included in this article. The complete verbatim chatbot responses are provided in Supplementary File S1. Additional question-level analytical values supporting the main findings are available from the corresponding author upon reasonable request. No patient-level or personally identifiable data were collected.

## Supplementary Materials

The following supporting information can be downloaded at https://www.mdpi.com/article/10.3390/healthcare14183119/s1. File S1: Complete Archived Chatbot Responses.

## Abbreviations

AI	Artificial Intelligence
ARI	Automated Readability Index
CHART	Chatbot Assessment Reporting Tool
CL	Coleman-Liau Index
EQIP	Ensuring Quality Information for Patients
FKGL	Flesch-Kincaid Grade Level
FRES	Flesch Reading Ease Score
GFI	Gunning Fog Index
GQS	Global Quality Score
JAMA	Journal of the American Medical Association
PA	Primary Aldosteronism
SMOG	Simple Measure of Gobbledygook
